# High external power narrow bandwidth erbium doped waveguide laser on thin film lithium niobate

**DOI:** 10.1515/nanoph-2025-0355

**Published:** 2025-11-03

**Authors:** Yu Ma, Mengqi Li, Jianping Yu, Zhiwei Fang, Min Wang, Jintian Lin, Haisu Zhang, Ya Cheng

**Affiliations:** State Key Laboratory of High Field Laser Physics and CAS Center for Excellence in Ultra-Intense Laser Science, Shanghai Institute of Optics and Fine Mechanics (SIOM), Chinese Academy of Sciences (CAS), Shanghai 201800, China; The Extreme Optoelectromechanics Laboratory (XXL), School of Physics and Electronic Sciences, 12655 East China Normal University, Shanghai 200241, China; State Key Laboratory of Precision Spectroscopy, 12655 East China Normal University, Shanghai 200062, China; Hefei National Laboratory, Hefei 230088, China; Shanghai Research Center for Quantum Sciences, Shanghai 201315, China; Collaborative Innovation Center of Extreme Optics, Shanxi University, Taiyuan 030006, China

**Keywords:** erbium-doped waveguide laser, thin-film lithium niobate, high-power narrow-bandwidth laser

## Abstract

Erbium-doped waveguide lasers have attracted great interests in recent years due to their compact footprint, high scalability and low phase noise. In this work, by using a high-external-gain erbium-doped thin-film lithium niobate waveguide as the gain medium, a fiber-Bragg-grating based Fabry–Perot-type laser is demonstrated with the low pump threshold of few-milliwatts, narrow bandwidth of 0.1 nm, high external output power above 2 mW and maximum optical signal-to-noise ratios above 50 dB. Laser linewidth measurements by self-delayed homodyne and heterodyne detections reveal the underlying multi-longitudinal-mode laser structure and the average intrinsic linewidth as low as ∼50 kHz for the individual longitudinal-modes. Theoretical modeling of the waveguide laser is also conducted with high consistence with the experimental measurements. The demonstrated high-power erbium-doped waveguide laser on thin-film lithium niobate can find diverse applications in optical communication and laser sensing.

## 1 Introduction

Erbium-doped waveguide lasers (EDWLs) are compact, efficient light sources that leverage erbium ions (Er^3+^) embedded in dielectric or semiconductor waveguides to generate laser emission covering the important telecom C-band (1,530–1,565 nm) wavelength range, ideal for telecommunications, sensing, and integrated photonics [1]. By combining the high gain of erbium-doped materials with the confinement and scalability of waveguide structures, EDWLs offer advantages such as low threshold power, narrow linewidth, and seamless on-chip integration [2], [3]. Recent advances in nanophotonic engineering, ion-doping strategies, and heterogeneous integration have further enhanced the attainable performance of EDWLs, enabling applications in optical communications, light detection and ranging (LiDAR), quantum photonics, and biomedical imaging [4], [5], [6].

Stimulated by the excellent nonlinear optical and electro/acousto-optical properties of the ferroelectric lithium niobate crystals, photonic integrated circuits (PICs) on the monocrystalline thin-film lithium niobate (TFLN) have been broadly investigated in the recent decade [7], [8], [9]. With the advent of the erbium-doped TFLN various kinds of lasers have been realized on this platform, including the micro-disk/ring resonator lasers, the on-chip Sagnac-reflector cavity lasers, and the photonic crystal nano-lasers [10], [11], [12], [13], [14], [15], [16], [17], [18], [19], [20], [21]. Compared to the established Er:Ti:LiNbO_3_ waveguide lasers developed in bulk LN crystals, the Er^3+^-TFLN lasers are much more compact with low thresholds [22], [23], [24], [25], [26]. However, due to the challenges of efficient light-cavity coupling and the extra cavity losses induced on-chip, the demonstrated Er^3+^-TFLN lasers are rather limited in the external output powers, i.e., the power transmitted into the output fibers or other optical components. As a result, the recent applications of Er^3+^-TFLN lasers necessitate the use of external optical amplifiers to boost the laser power to milliwatt-levels.

In this work, by adopting a high-external-gain Er^3+^-TFLN waveguide as the laser gain element, a Fabry–Perot-type linear cavity laser is built by adding two fiber Bragg gratings (FBGs) connected to both ends of the waveguide with bidirectional pumping using ∼1,480 nm laser diodes. The lasing properties of the FBG-assisted waveguide laser are systematically investigated, giving the low laser thresholds of few-milliwatts coupled pump powers, the maximum laser output power of 2.67 mW and the maximum optical signal-to-noise ratio exceeding 50 dB. Besides, the laser integral linewidth is less than 0.1 nm with multi-longitudinal-modes spaced by ∼80 MHz as revealed by the self-delayed homodyne detection. The average intrinsic linewidth of the single-longitudinal-mode is also measured as low as ∼50 kHz by the self-delayed heterodyne detection. Theoretical modeling of the waveguide laser using the erbium rate equations and the boundary conditions at both ends of the laser cavity predict similar lasing properties in high consistence with the experiment measurements. The demonstrated high-power Er^3+^-TFLN waveguide laser can be further integrated with high-speed electro-optical modulation element for fast wavelength tuning, holding great promise in optical communication and laser sensing.

## 2 Device design and fabrication

**Figure 1:**
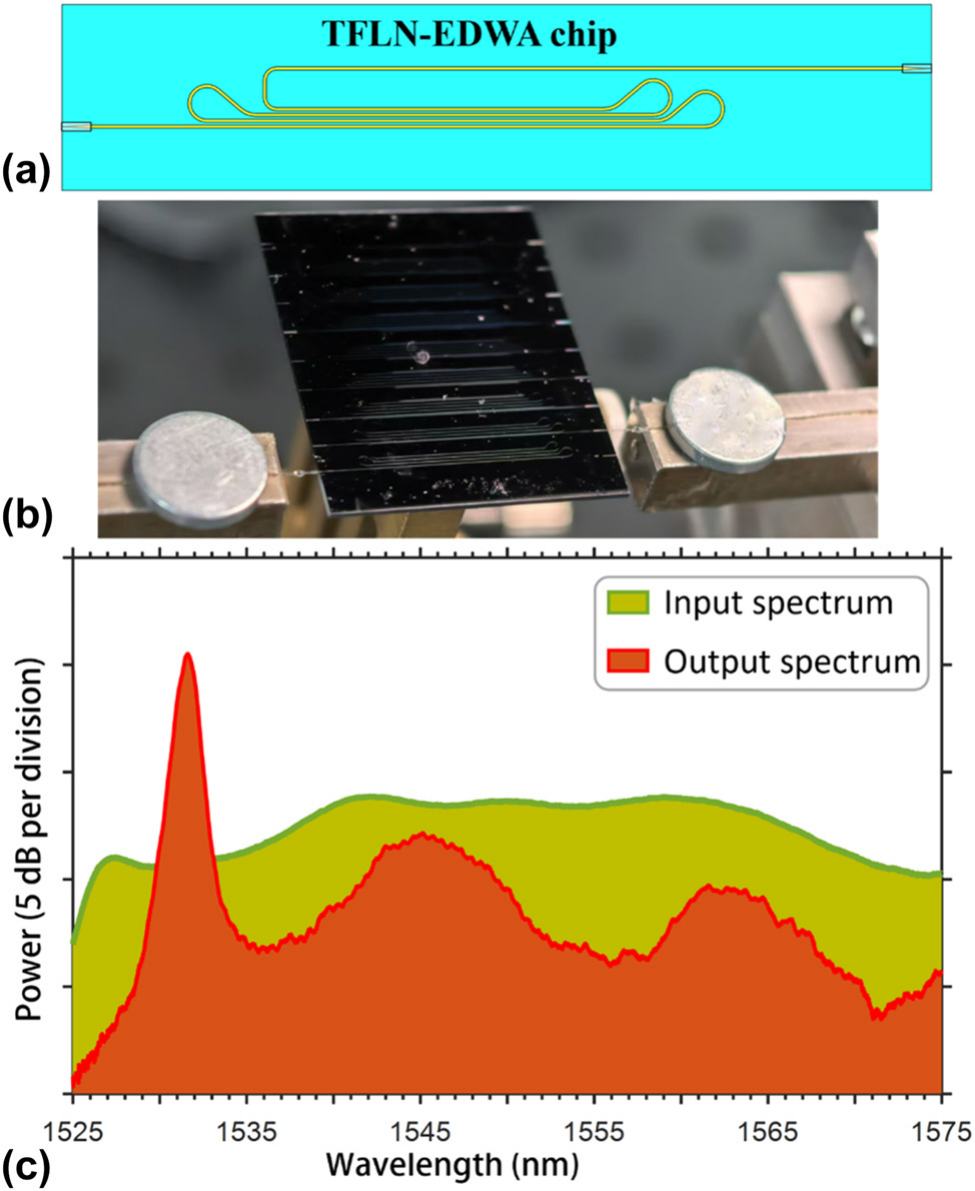
Er^3+^-doped TFLN waveguide chip. (a) Schematic diagram of the erbium-doped thin-film lithium niobate waveguide amplifier chip. (b) Photograph of the fabricated amplifier chip coupled with optical fibers. (c) Typical external gain spectra of the amplifier chip.

The Er^3+^-TFLN waveguide chip is built on the erbium-doped Z-cut lithium niobate on insulator (LNOI) wafer prepared by the ion-slicing process (NanoLN, Jinan Jingzheng) from the congruent erbium-doped lithium niobate bulk crystal. The erbium-doping concentration is around 0.5 mol%. The top LN layer thickness is 500 nm, and the buried oxide layer and the bottom silicon substrate are 4.7-μm-thick and 500-μm-thick, respectively. The TFLN waveguide is fabricated by the photolithography assisted chemo-mechanical etching (PLACE) technique which consists of femtosecond laser patterning and chemo-mechanical polishing. The fabrication details can be found in our previous works [27], [28]. The footprint of the designed waveguide chip is shown in Figure 1(a). Waveguide bends with minimum radius of 400 μm are employed to reduce the size of the chip and the total waveguide length is 8.5 cm. Besides, at both ends of the Er^3+^-TFLN waveguide index-matched edge couplers are integrated for high-efficiency coupling with optical fibers. The edge couplers are based on the spot-size-convertor (SSC) allowing adiabatic mode conversion from the underneath TFLN waveguide to the overcladding SiON waveguide. Detailed information about the SSC edge couplers can be found in previous works [29], [30].

The fabricated Er^3+^-TFLN waveguide chip is shown in Figure 1(b), where the chip is butt-coupled with optical fibers from both ends. The employed coupling fibers are the ultrahigh-NA fiber (UHNA7), which has the NA = 0.41 and a mode field diameter (MFD) of 3.2 μm at 1,550 nm. The UHNA7 fiber is fusion-spliced with standard single mode fiber (SMF), which is then connected to other fiber modules like FBG and fiber-coupled laser diodes by flange connectors. The facet coupling loss between the UHNA7 fiber and the waveguide edge coupler is measured to be less than 2 dB at 1,550 nm, while the splicing loss between the UHNA7 fiber and the SMF fiber is around 1 dB. Thus, the lumped coupling loss from the SMF to the chip is around 3 dB/facet. The external (fiber to fiber) gain properties of the Er^3+^-TFLN waveguide are tested by launching a broadband light from the amplified spontaneous emission (ASE) source into the chip simultaneously pumped by 1,480 nm laser diodes. The typical gain spectra are shown in Figure 1(c), where the yellow-shaded region denotes the input power spectrum and the red-shaded region shows the output power spectrum. It can be clearly seen that the gain is only remarkable around the peak wavelength of 1,532 nm, so the central wavelength of the FBG used for external laser generation is selected in accordance with the gain peak of the Er^3+^-TFLN waveguide chip to utilize the maximum gain capacity.

## 3 Results and discussion

**Figure 2:**
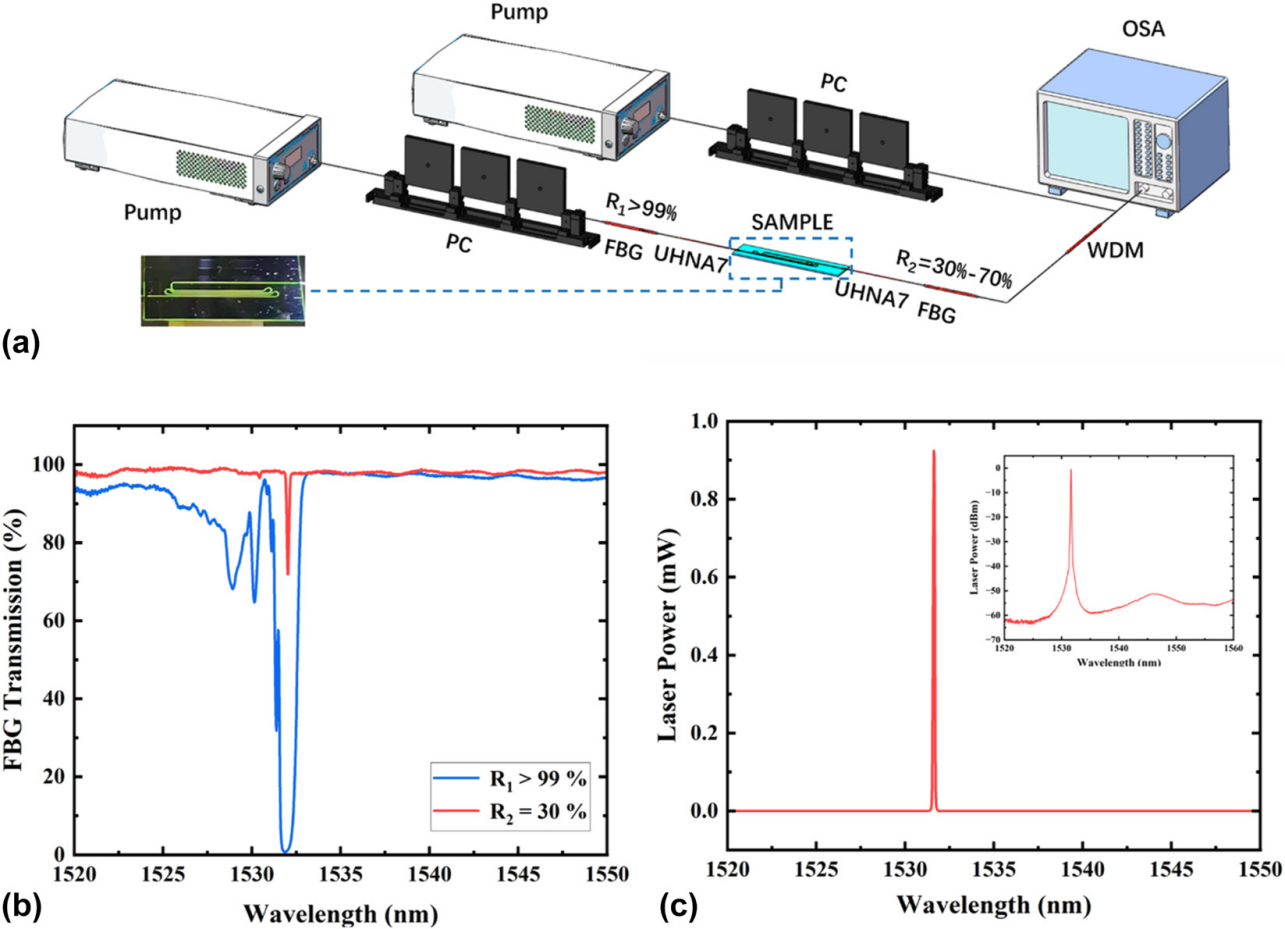
FBG-assisted TFLN-waveguide laser. (a) Experimental schematic for characterizing the EDWA-based laser, and the inset image is the photograph of the EDWA chip showing bright upconversion fluorescence during the test process (pump: laser diodes at the wavelength of 1,480 nm; PC: polarization controller; FBG: fiber Bragg gratings; UHNA7: ultra-high-NA fiber of NA = 0.41; WDM: wavelength division multiplexer; OSA: optical spectrum analyzer). (b) Transmission spectra of FBGs with *R*_1_ > 99 % and *R*_2_ = 30 % used at both sides of the chip in the experiment. (c) Laser output spectrum measured by OSA in linear scales, and the inset is the laser spectrum in logarithmic scales.

The experimental setup for characterization of the FBG-assisted Er^3+^-TFLN waveguide laser is shown in Figure 2(a). Two FBGs of different reflection ratios are employed at both ends of the chip. The high-reflection FBG with *R*_1_ > 99 % is connected to the left side, while the FBG of variable reflection ratios between 30 % and 70 % is put on the right side in order to facilitate the output of the intra-cavity laser power. Fiber-based 1,480 nm/1,550 nm wavelength division multiplexer (WDM) and polarization controller (PC) are inserted after the FBGs in both sides, in order to pump the waveguide chip from both directions by two high-power 1,480 nm laser diodes. The laser output spectra are measured after the right WDM by the optical spectrum analyzer (OSA), which is pre-calibrated by a high-precision power meter.

The transmission spectral profiles of the two FBGs are shown in Figure 2(b). It can be seen that the reflection bandwidth of the *R*_1_ > 99 % FBG is larger than 1 nm, while the bandwidth of the *R*_2_ = 30 % FBG is around 0.1 nm. The central wavelengths of both FBGs are close to the desired wavelength of 1,532 nm. By increasing the pump powers an intense sharp line gradually emerges on the broadband amplified spontaneous emission pedestal of erbium ions, and the generated laser peak around 1,532 nm is clearly observable with a typical laser spectrum shown in Figure 2(c).

**Figure 3:**
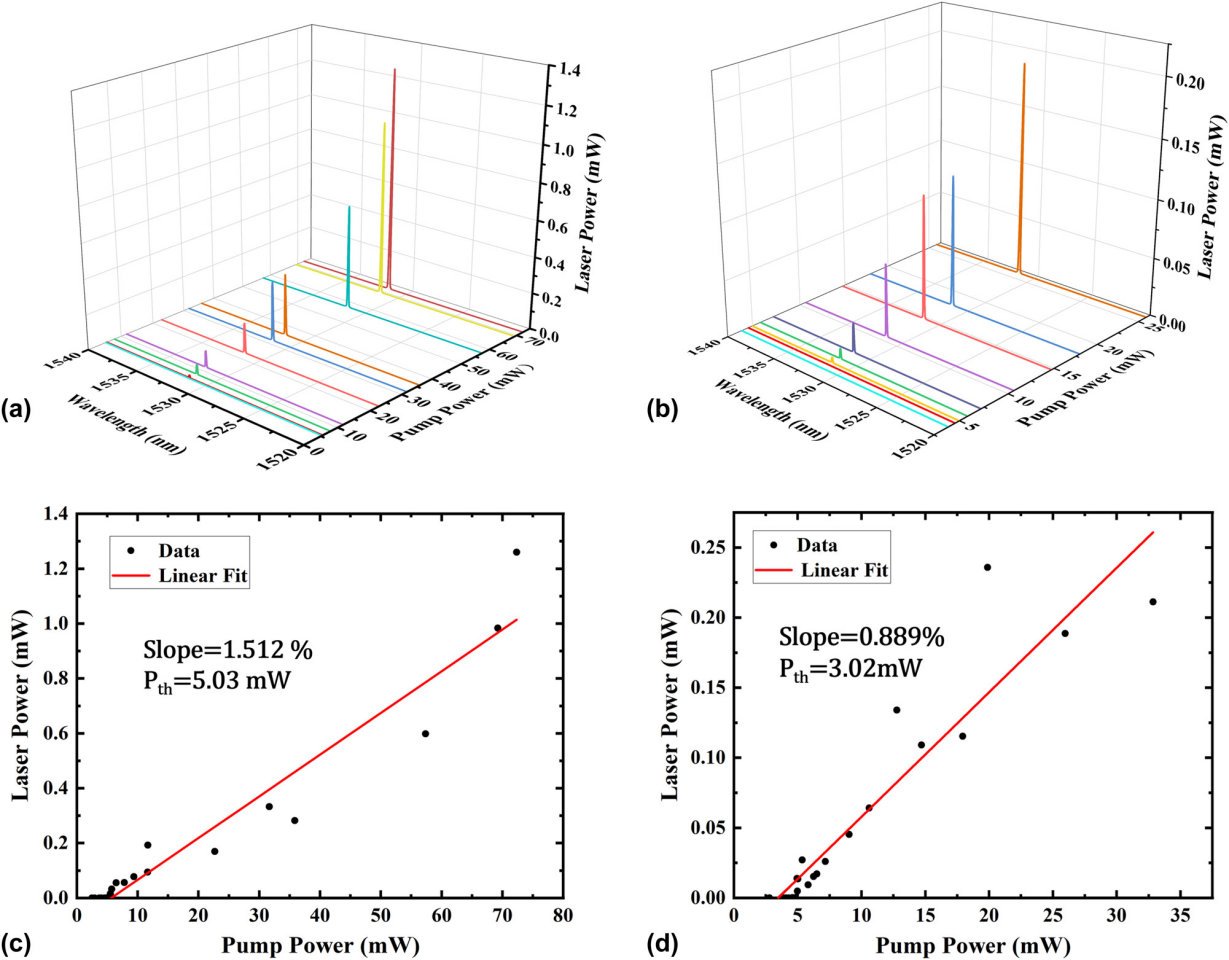
Waveguide laser characterization. (a) and (b) Show the evolution of the induced laser spectrum with the pump power when the reflectance of the FBG at the rear of the sample is *R*_2_ = 30 % and *R*_2_ = 70 %, respectively. (c) and (d) Show the variation of laser power with increasing pump power when the reflectance of the FBG at the rear of the sample is *R*_2_ = 30 % and *R*_2_ = 70 %, respectively. The laser slope efficiency and the threshold power are also marked in the figures (c) and (d), respectively.

The lasing properties of the FBG-assisted waveguide laser are further investigated at increasing pump powers and variable output reflection ratios. The laser spectral evolution with coupled pump powers at the cavity output conditions of *R*_2_ = 30 % and *R*_2_ = 70 % are measured and shown in Figure 3(a) and (b), respectively. The measured laser linewidths by OSA are around 0.1 nm, which is limited by the minimum OSA resolution of 0.05 nm. The linewidth measured on OSA should be the integral bandwidth of multi-longitudinal-modes as revealed by the high-resolution self-beating detections conducted later. It can be clearly seen from Figure 3(a) and (b) that the lasing emissions set in at comparable low pump powers of few milliwatts. The measured laser powers at increasing pump powers are further plotted in Figure 3(c) and (d), from which the laser threshold and slope efficiency can be easily fitted. For the case of lasing at *R*_2_ = 30 %, the threshold pump power is found to be around 5.03 mW, and the slope efficiency is deduced as 1.512 %. Similarly, when *R*_2_ = 70 %, the lasing threshold and the slope efficiency are deduced as 3.02 mW and 0.889 %.

The loss of the FBG-assisted waveguide laser mainly consists of the on-chip loss of the gain chip, the reflection loss of the resonant cavity composed of FBGs, and the coupling loss from the fiber to the gain chip. By setting the coupling loss as *α* and the on-chip net gain as *G*_0_ (both in the units of dB), the threshold condition for lasing is that the net gain equals the total loss for the round-trip light pass as:

(1) $$2G_{0}=4\alpha +\log_{10}\left(1-R_{1}\right)\left(1-R_{2}\right)$$

The reflectance *R*_1_ and *R*_2_ of FBG will affect the laser threshold power. As can be seen from the above formula, the larger *R*_2_ is, the smaller the threshold power will be. So, the threshold power when *R*_2_ = 70 % (3.02 mW) is less than that when *R*_2_ = 30 % (5.03 mW). Meanwhile, when *R*_2_ increases, the transmittance *T*_2_ = 1 − *R*_2_ decreases, indicating that the output power will decrease, and the proportion of the output power to the total loss will decrease, and the slope efficiency proportional to it will also decrease. So, the slope efficiency when *R*_2_ = 70 % (0.889 %) is lower than that when *R*_2_ = 30 % (1.512 %).

**Figure 4:**
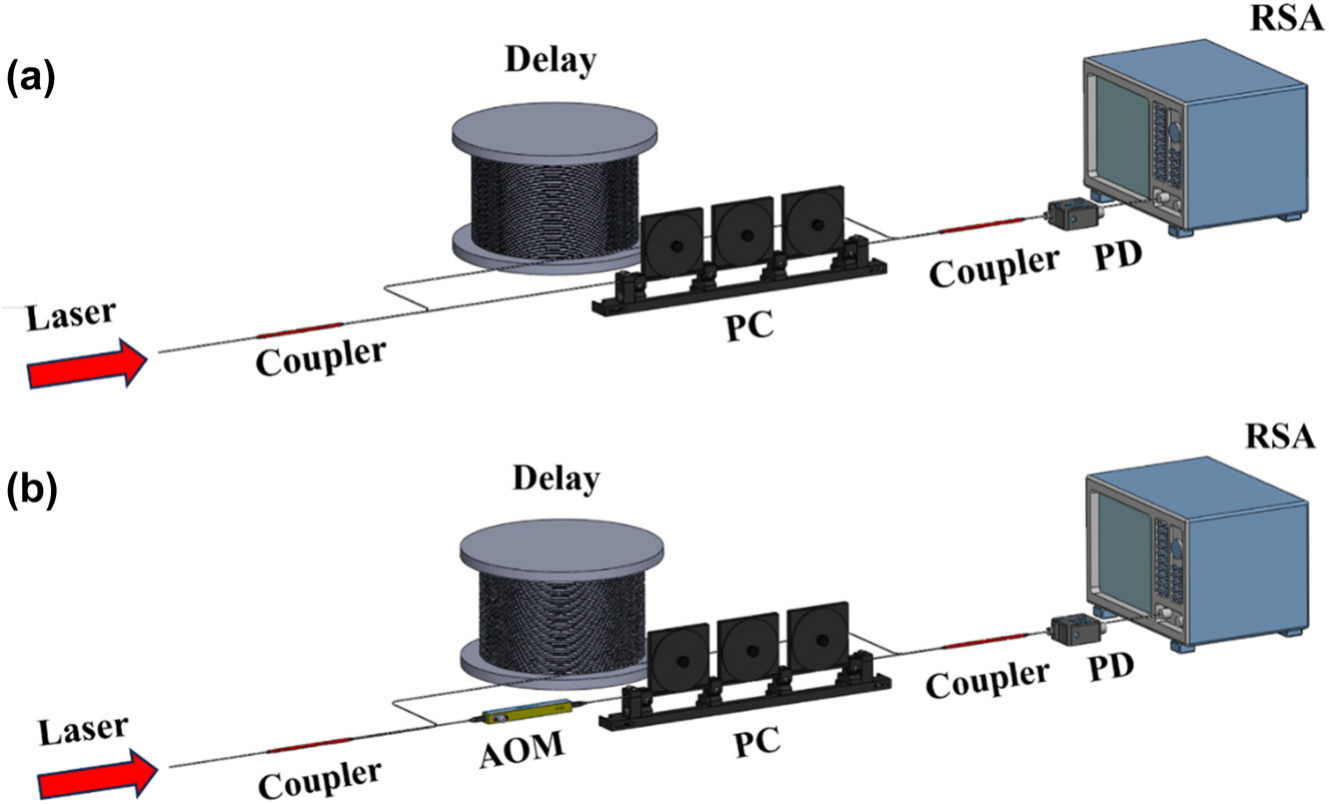
Laser linewidth characterization. (a) Experimental setup used to measure the longitudinal mode spectrum of the output laser by delayed self-homodyne method (delay: 5-km-long single-mode optical fiber; PC: polarization controller; PD: photodetector; RSA: real-time spectrum analyzer). (b) Experimental setup used to measure the intrinsic single-mode linewidth of the output laser by delayed self-heterodyne method (AOM: acousto-optic modulator with a modulation frequency of 100 MHz).

Due to the long fiber length (∼1 m) in the FBG-SMF-UNHA7-chip connection link, the effective cavity length of the laser is much larger than the Er^3+^-TFLN waveguide length (8.5 cm). Therefore, the longitudinal mode spacing for such long laser cavity is estimated to be Δ*ν* = *c*/2*n*_*g*_ *L*, where *n*_*g*_ is the fiber group index at the laser wavelength and *L* is the single-pass length of the laser cavity. Using *n*_*g*_ = 1.5 for the optical fiber and *L* = 1 m the longitudinal mode spacing is estimated to be Δ*ν* = 100 MHz. Thus, under the reflection bandwidth of the output FBG coupler (*R*_2_ = 30 %), about 100 longitudinal modes exist and multi-longitudinal-mode lasing is expected under the current cavity lengths. To identify the longitudinal modes of the generated laser at the 100 MHz-level spacing the self-delayed homodyne detection is employed [31]. The experimental setup is shown in Figure 4(a). The FBG-assisted waveguide laser is first split into two paths by a 50:50 fiber coupler. One light path passed through the polarization controller, while the other path was delayed by the single-mode fiber with the length of 5 km. Then the two light paths are combined with another 50:50 fiber coupler and the beating signals are measured by the fast photo-detector (PD) whose output signals are further analyzed by the electrical real-time spectrum analyzer (RSA, Tektronix RSA5126B). Besides, a similar self-delayed heterodyne detection is also employed to identify the intrinsic linewidth of the individual longitudinal mode. The experimental setup for heterodyne detection is shown in Figure 4(b), with the only difference with the homodyne detection by inserting an acousto-optic modulator (AOM, AOM-1550-200M, Csrayzer Inc.) on the first light path which shifted the light frequency by 100 MHz.

**Figure 5:**
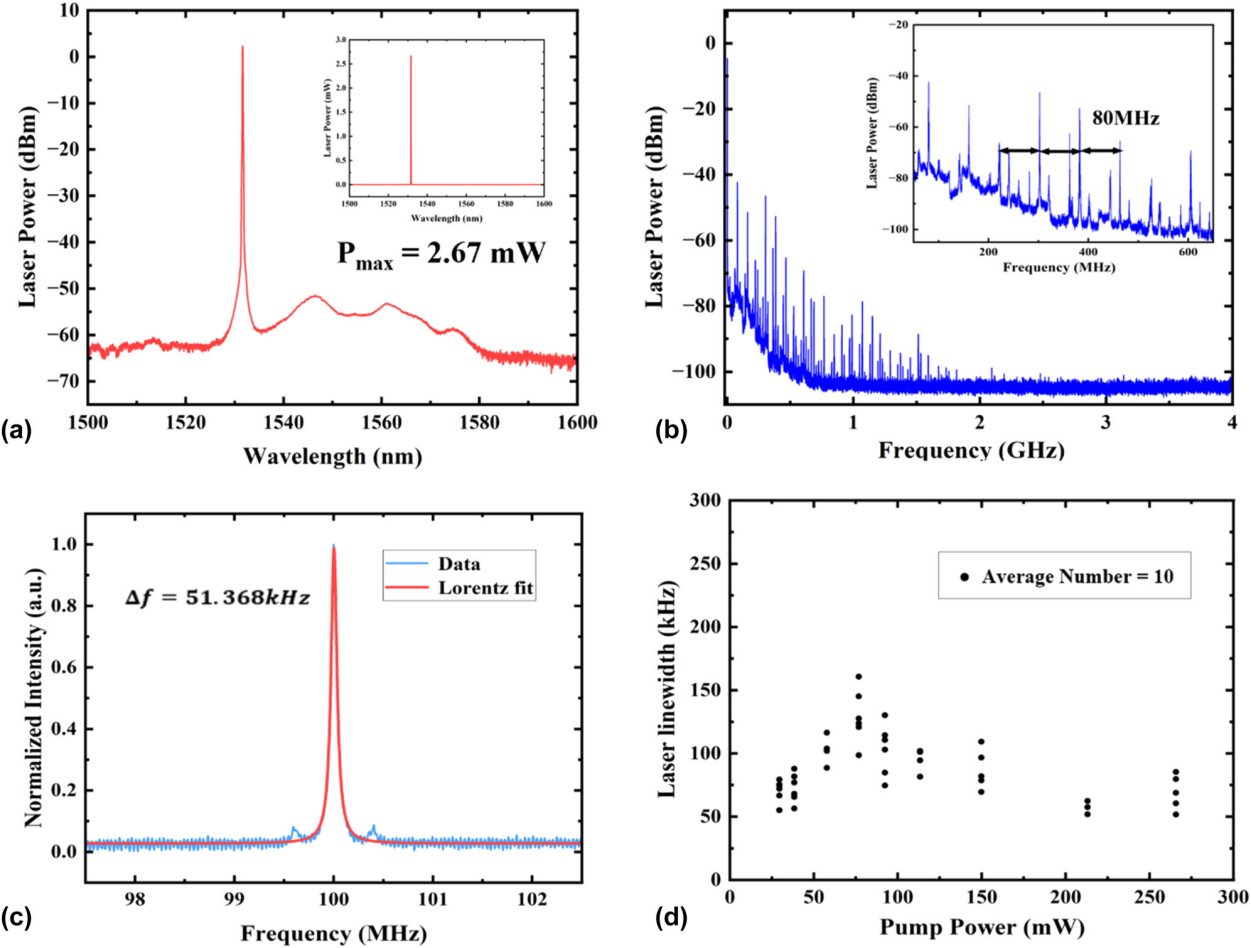
Linewidth characterization results. (a) Laser spectrum at the maximum output power in logarithmic scales, and the inset figure shows the laser output spectrum in linear scales giving the maximum laser of 2.67 mW. (b) Self-homodyned beat spectrum of the laser showing the multi-longitudinal-mode profiles. The retrieved longitudinal mode spacing is 80 MHz. (c) Self-heterodyned beat spectrum of the laser showing the average intrinsic single-longitudinal-mode linewidth of 51.368 kHz by Lorentz fitting. (d) Variations of the average single-longitudinal-mode linewidth under different pump powers. The average number of each point is 10.

Before testing the waveguide laser linewidth by the above setups, the attainable laser output powers are examined by increasing the bidirectional pump powers above 200 mW. The maximum laser output power is 2.67 mW with the corresponding spectrum shown in Figure 5(a), from which the optical signal to noise ratio (OSNR) can be obtained by comparing the laser peak-level with the background ASE-pedestals to be above 50 dB. The high-external-output power of the demonstrated waveguide laser enables the direct laser beating measurement by the photo-diode without using the external optical amplifiers as commonly employed in the laser linewidth measurements of Er^3+^-TFLN lasers. The measured homodyne beating signals are shown in Figure 5(b), where multiple beating peaks with frequency separation around 80 MHz extending until the cutoff frequency about 2 GHz are clearly seen. The measured multiple beating signals of the waveguide laser should reflect its multi-longitudinal-mode lasing structure. Besides, there also exist weak beating peaks within adjacent main beating peaks, which are ascribed to the higher-order transverse modes excited in the Er^3+^-TFLN waveguide having different propagation constants and thus laser resonance structures compared to the fundamental mode.

The intrinsic linewidth of individual longitudinal mode is further measured by the self-delayed heterodyne detection and the beat signal at the central frequency of 100 MHz (the shift frequency induced by the AOM) is shown in Figure 5(c). Due to the multi-longitudinal-mode structure of the laser, the measured beating spectrum at the AOM shift frequency should be the averaged signals over self-heterodyne beating of multiple longitudinal modes. Following a Lorentzian fit, the average intrinsic linewidth of the individual longitudinal modes of the laser is deduced to be 51.368 kHz. The stability of the laser’s intrinsic linewidth is further examined by multiple measurements under different pump powers. The experimental data are shown in Figure 5(d), with each data point representing the average of 10 measurements. It can be observed that as the pump power varies, the average intrinsic linewidths of individual longitudinal mode remain within the range of 50–150 kHz. Future work will concentrate on selecting single-longitudinal-mode in the laser cavity by shortening the cavity length and adding mode-selective filters to obtain single-mode waveguide laser with comparable linewidths.

**Figure 6:**
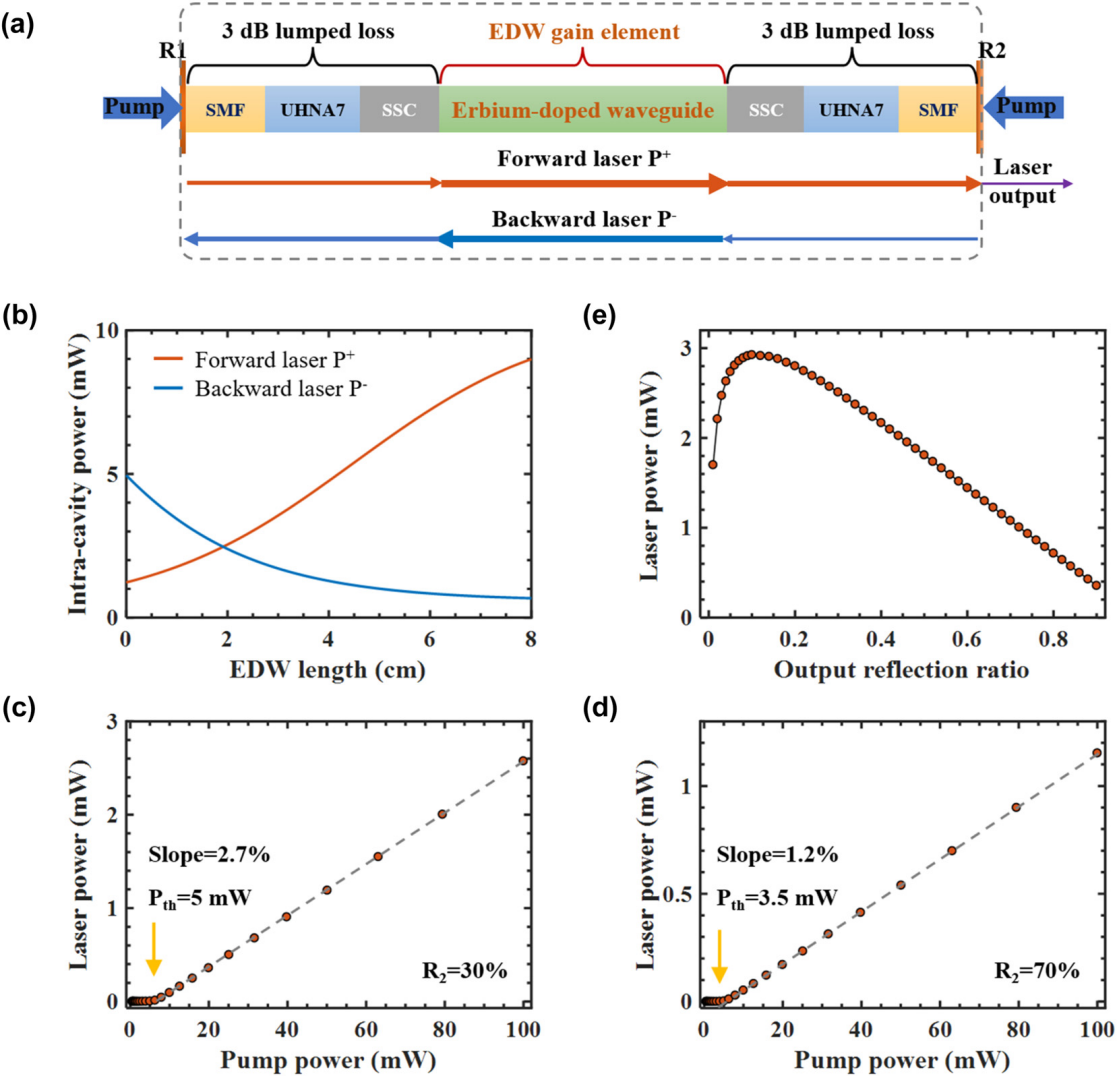
Waveguide laser simulation results. (a) Schematic diagram of the FBG-EDWA laser (SMF: single mode fiber, UHNA7: ultraHigh-NA fiber, SSC: spot size convertor, *R*_1_ & *R*_2_: reflection ratios of the two FBG at both cavity ends). (b) Simulated intra-cavity laser power evolution. (c) and (d) Simulated laser power variations with increasing pump powers for *R*_2_ = 30 % and *R*_2_ = 70 %, respectively. The retrieved laser thresholds and slope efficiencies are labelled in both figures. (e) Simulated laser power variations with different reflection ratios *R*_2_ at the output end.

To understand the lasing properties of the waveguide laser, a theoretical model including the gain dynamics of the Er^3+^-TFLN waveguide and the output coupling conditions of the laser cavity is employed [32]. The schematic diagram of the waveguide laser used in the simulation is shown in Figure 6(a). The intracavity forward and backward laser powers are denoted as *P* ^+^ and *P* ^−^, respectively. The laser signals circulating in the cavity will experience coupling losses at the fiber-chip interface and being reflected at the two FBGs, and then amplified by the Er^3+^-TFLN waveguide. The waveguide gain is described by the static population rate equations of erbium ions in-band pumped by the 1,480 nm lasers under two-level approximation (^4^I_15/2_ and ^4^I_13/2_). To initialize the lasing process the spontaneous emission noise peaked at the laser wavelength is added in the simulation [33]. The bidirectional pump powers and laser powers are propagated in the cavity under the boundary conditions for the intracavity laser powers as:

(2) $$P^{+}\left(0\right)=\alpha^{2}R_{1}P^{-}\left(0\right)$$

(3) $$P^{-}\left(L\right)=\alpha^{2}R_{2}P^{+}\left(L\right)$$

where $P^{\pm }\left(0\right)$ denote the intracavity laser powers at the left end of the gain waveguide, and $P^{\pm }\left(L\right)$ denote the intracavity laser powers at the right end of the gain waveguide. *α* = 50 % represents the lumped coupling loss (3 dB) between the SMF and the waveguide chip, *R*_1_ = 99 % for the left FBG reflector and *R*_2_ = 30 % for the right FBG reflector. *L* is the length of gain waveguide which is set to *L* = 8 cm in the simulation by subtracting the lengths of SSC edge couplers at both ends. The output laser power is obtained by $P_{\text{out}}=\alpha \left(1-R_{2}-\alpha_{s}\right)P^{+}\left(L\right)$ where *α*_*s*_ = 5 % is the transmission loss of the output FBG.

The simulated intracavity laser power evolution is shown in Figure 6(b). The launched pump power in the Er^3+^-TFLN waveguide is 100 mW. The intracavity laser powers are reduced at both the waveguide ends due to coupling losses and FBG reflections, and then amplified when propagating along the waveguide. Due to the high output coupling ratio the intracavity forward laser power is higher than the backward laser power to maintain gain–loss balance. The dependence of laser powers on the launched pump powers are further simulated with *R*_2_ = 30 % and *R*_2_ = 70 %, with the results shown in Figure 6(c) and (d), respectively. It can be easily noticed the threshold pump powers for lasing in both cases are close to the experimental measurement. Besides, the simulated laser slope efficiencies of 2.7 % and 1.2 % are qualitatively consistent with the measured slope efficiencies of 1.512 % and 0.889 % for *R*_2_ = 30 % and *R*_2_ = 70 %, respectively. The measured lower slope efficiencies than the simulated values may come from excited-state absorption and cooperative upconversion of erbium ions which is not included in the gain model. The laser output powers at variable output FBG reflection ratios are further simulated with *R*_2_ changing from 5 % to 90 %. The optimal output reflection ratio shown in Figure 6(e) is about 10 %, where the laser power is close to 3 mW. Such reflection ratio is comparable to the Fresnel reflection ratio of TFLN waveguide in air. The simulated output power at *R*_2_ = 30 % is about 2.7 mW, which is also close to the measured maximum laser power of 2.67 mW. Future works by reducing the coupling losses and increase the gain waveguide length can be employed to elevate the Er^3+^-TFLN waveguide laser power to higher levels, and nonlinear effects at high laser powers in the Er^3+^-TFLN waveguide such as excited state absorption, photorefractive index change and modal instability should be paid attention considering the strong modal confinement.

## 4 Conclusions

In conclusion, a high-external-power narrow bandwidth Er^3+^-TFLN waveguide laser at the central wavelength around 1,532 nm has been realized in this work. By combining the high-gain Er^3+^-TFLN waveguide with external FBG reflectors a Fabry-Perot-type linear laser cavity is built with easy operation. The threshold pump power and laser slope efficiency of the FBG-assisted waveguide laser are measured as 5.03 mW and 1.512 % for output FBG reflection of 30 %, 3.02 mW and 0.889 % for output FBG reflection of 70 %, respectively. The maximum transmitted laser power into the output fiber is 2.67 mW, and the laser bandwidth is less than 0.1 nm with multi-longitudinal-modes spaced by 80 MHz. The average intrinsic linewidth of the individual longitudinal modes is measured to be as low as ∼50 kHz. The demonstrated Er^3+^-TFLN waveguide laser holds great promise in optical communication and laser sensing.
